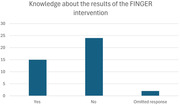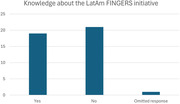# Survey on knowledge of Uruguayan neurologist about the FINGER intervention

**DOI:** 10.1002/alz.095641

**Published:** 2025-01-09

**Authors:** Valeria Contreras, Ana Charamelo, Sergio Dansilio

**Affiliations:** ^1^ LatAm FINGERS, Montevideo, Montevideo Uruguay; ^2^ Hospital Británico, Montevideo, Montevideo Uruguay

## Abstract

**Background:**

The Latin American Initiative for Lifestyle Intervention to Prevent Cognitive Decline (LatAm‐FINGERS) is based on the study FINGER (Finnish Geriatric Intervention Study to Prevent Cognitive Impairment and Disability) in which participate 12 Latin American countries. Is the first non‐pharmacological multicenter randomized clinical trial to prevent cognitive impairment in Latin America (LA), and investigates the feasibility of a multi‐domain lifestyle intervention and the efficacy of the intervention in LA.

We were interested in knowing what information Uruguayan neurologists have on the subject. In Uruguay, according to official information, in 2021 there were 139 registered neurologists. No surveys have been carried out on the subject in Uruguay.

Objectives: To know what information the Uruguayan neurologists have about the results of the FINGER intervention and the frequency of indication of primary prevention measures to people at risk of cognitive impairment.

**Methods:**

This is a cross sectional, observational and descriptive trial. A survey was designed in the SurveyMonkey application with 13 questions and it was sent to neurologists through social networks. Informed consent was requested.

**Results:**

The survey was answered by 41 neurologists. 38.5% of responders were aware of the results of the FINGER intervention in Finland and 36% have made prevention recommendations taking them into consideration.

40 participants responded regarding primary prevention measures. Of these, 90% stated that they made recommendations to people at risk of cognitive impairment. 30% reported making nutritional suggestions frequently, 95% suggested cognitive stimulation exercises and 92.5% suggested participating in regulated physical activity programs.

47.5% knew about the LatAm FINGERS initiative and 42.5% knew that Uruguay was participating in it. All respondents expressed interest in knowing the results of the LatAm FINGERS initiative when it has concluded.

**Conclusions:**

Most Uruguayan neurologists make recommendations on primary prevention measures for cognitive impairment. There was moderate knowledge about the results of the FINGERS study, but there is a marked interest in learning more about the topic.

It is planned to extend the survey to other medical specialists involved in the care of people at risk of cognitive impairment.